# Atrioventricular block of intraoperative device closure perimembranous ventricular septal defects; a serious complication

**DOI:** 10.1186/1471-2261-12-21

**Published:** 2012-03-29

**Authors:** Qiang Chen, Hua Cao, Gui-Can Zhang, Liang-Wan Chen, Qian-Zhen Li, Zhi-Huang Qiu

**Affiliations:** 1 Department of Cardiovascular Surgery, Union Hospital, Fujian Medical University, Xinquan Road 29#, Gulou District, Fuzhou 350001, People's Republic of China

**Keywords:** CHD, Septal defects, Minimally invasive, Atrioventricular block

## Abstract

**Background:**

Atrioventricular block (AVB) is a well-reported complication after closure of perimembranous ventricular septal defects (VSDs). To report the occurrence of AVB either during or following closure of perimembranous VSDs using a novel "hybrid" method involving a minimal inferior median incision and of intraoperative device closure of the perimembranous VSDs.

**Methods:**

Between January 2009 and January 2011, patients diagnosed with perimembranous VSDs eligible for intraoperative device closure with a domestic occluder were identified. All patients were assessed by real-time transesophageal echocardiography (TEE) and electrocardiography.

**Results:**

Of the 97 included patients, 94 were successfully occluded using this approach. Complete AVB occurred in only one case and one case of Mobitz type II AVB was diagnosed intraoperatively. In both patients, the procedure was aborted and the AVBs quickly resolved. Glucocorticosteroids were administered to another two patients who developed Mobitz type II AVB intraoperatively. Those two patients converted to Mobitz type I AVB 3 days and 5 days postsurgically. During the follow-up period (range, 6-24 months), one patient developed complete AVB 1 week following device insertion. Surgical device removal was followed by a rapid and complete recovery of atrioventricular conduction.

**Conclusions:**

Intraoperative device closure of perimembranous VSDs with a domestic occluder resulted in excellent closure rates; however, AVB is a serious complication that can occur either during or any time after device closure of perimembranous VSDs. The technique described herein may reduce the incidence of perioperative AVB complications. Surgeons are encouraged to closely monitor all patients postsurgically to ensure AVB does not occur in their patients. Additional long-term data to better identify the prevalence and risk factors for AVB in treated patients are needed.

## Background

Ventricular septal defects (VSD) are one of the most common congenital cardiac lesions. In the majority of cases (approximately 70%), VSDs are located in the area of the perimembranous region wedged between the tricuspid and aortic valves [1]. As an alternative to traditional surgical closure techniques, transcatheter closure of VSDs has gradually matured, especially since the introduction of the Amplatzer occlude. Transcatheter closure is minimally invasive, offers a good cosmetic outcome, and is often more acceptable to patients and their families than traditional surgical approaches [2-5].

Short- and medium-term follow-up data indicate that many of the shortcomings associated with device closures have been addressed. In those reports, the most significant complication was a transient arrhythmia. In patients with perimembranous VSDs treated via transcatheter closure, complete atrioventricular block (AVB) has been reported [6-10]. The purposes of this article were to report the incidence of AVB both during and after intraoperative device closure of perimembranous VSDs with a domestic occluder and the outcome of patients who experienced this complication.

## Methods

### Patients

Between January 2009 and January 2011, patients diagnosed with perimembranous VSDs undergoing intraoperative device closure with a domestic VSD occluder were identified. All patients with perimembranous VSDs measuring 5-12 mm in diameter with a rim > 2 mm away from nearby major cardiac structures (such as the aortic, tricuspid, and pulmonary valves) and no evidence of aortic regurgitation were included. Patients eligible for intraoperative device closure had hemodynamically significant left-to-right shunts, significant chamber enlargement, mild-moderate pulmonary hypertension despite medical therapy, and a history of infective endocarditis. Patients with VSDs in other locations or with coexisting cardiac anomalies were excluded. Routine preoperative examinations included a standard electrocardiogram, thoracic radiographs, and blood tests.

### Devices

**Figure 1:**
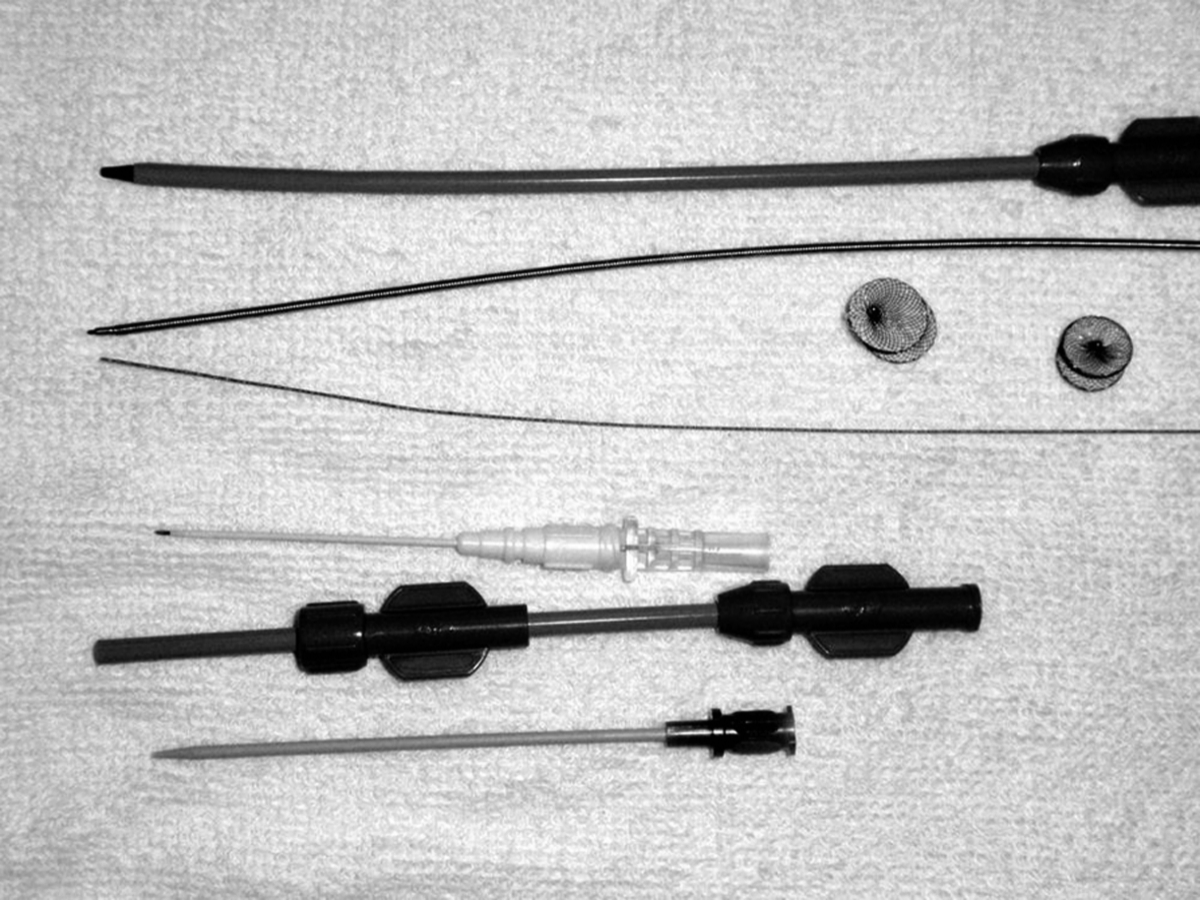
**The asymmetric device, the symmetric device, and the entire delivery system**.

The device delivery system consists of a trocar, guide wire, a dilator and delivery sheath, and a loading sheath made of polyethylene and acrylonitrile butadiene styrene and disinfected with ethylene oxide. The VSD occluder is a self-expandable, double-disk device. (Lifetech Scientific (Shenzhen) Co, Ltd and Shanghai Shape Memory Alloy Co, Ltd., China) As shown in Figure 1, both asymmetric and symmetric occluders were employed. On the left ventricular side of the asymmetric device, the aortic end of the disk is 1 mm wider than the waist to avoid impinging the aortic valve whereas the other side is positioned to be 5-6 mm wider than the waist and has a platinum marker to guide device orientation. For the symmetric device, the left ventricular disk is 2 mm larger than the waist. The right ventricular disk is 2 mm larger than the waist in both occluders. The occluders are sized according to the waist diameters, which range from 4 mm to 16 mm in 1 mm increments. The occluders were implanted using a 6-9 F delivery sheath.

### Protocol

In all patient the presence of a VSD was confirmed preprocedurally using transthoracic echocardiography (TTE) with color Doppler from the subxyphoid, apical, and parasternal views. During device deployment, TEE (GE vivid 7, GE Medical Systems, Milwaukee, WI, USA) was used for accurate determination of the VSD location, morphologic features, size (the largest diameter), and for visualization of the rims. The diameter of the VSD was measured by TTE using two-dimensional imaging and color flow Doppler on both the long and short axis views. General anesthesia was induced in all patients, who were subsequently placed in a supine position and draped for exposure to the entire thoracic cavity. Intraoperative TEE was used to assess the perimembranous VSD, particularly the defect size and circumferential margins adjacent to the aortic and tricuspid valves. An appropriately sized occluder was selected based on the largest size of the defect on TEE color Doppler imaging. Where the defect was aneurysmal or fenestrated, the maximum size of the mouth of the aneurysm (true VSD) was recorded as well as the exit point of the main defect into the right ventricle. The asymmetric device had a margin 0-2 mm in excess of the aortic rim.

**Figure 2:**
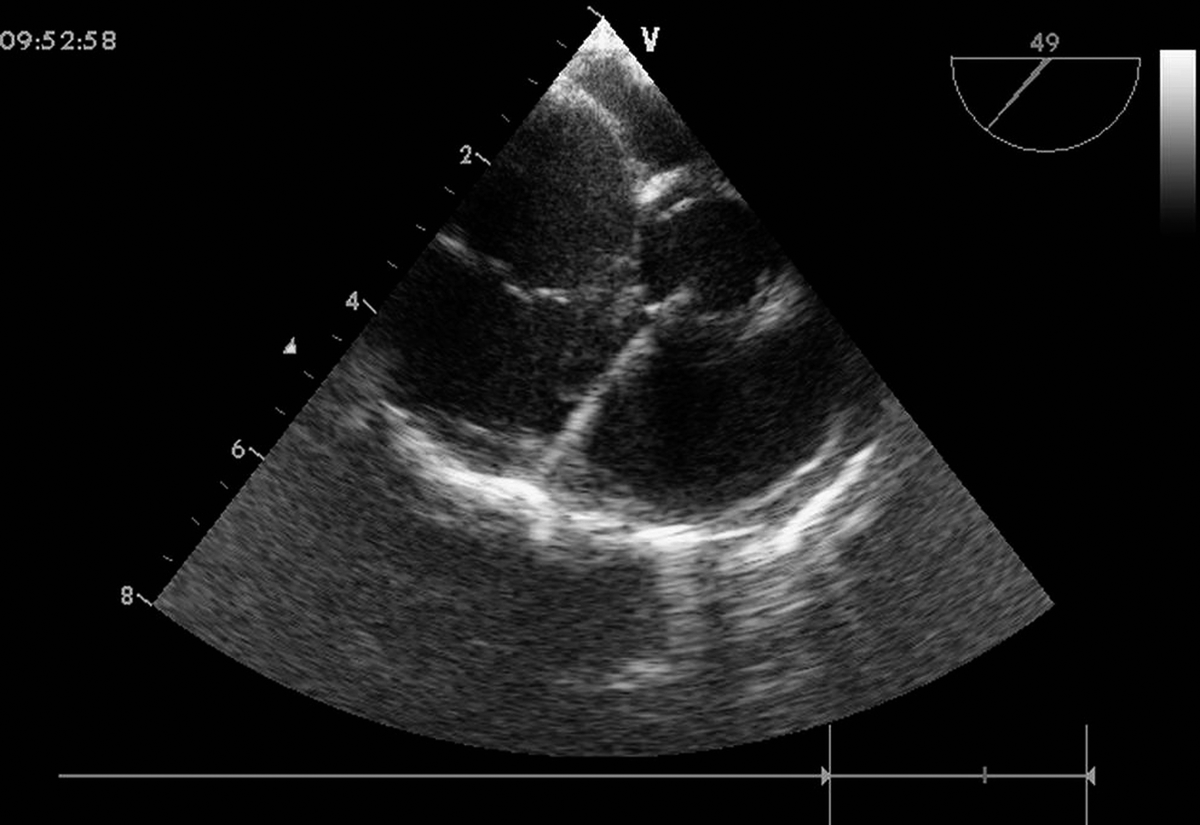
**A floppy wire positioned from the right ventricle free wall into the left ventricle cavity across the VSD**.

**Figure 3:**
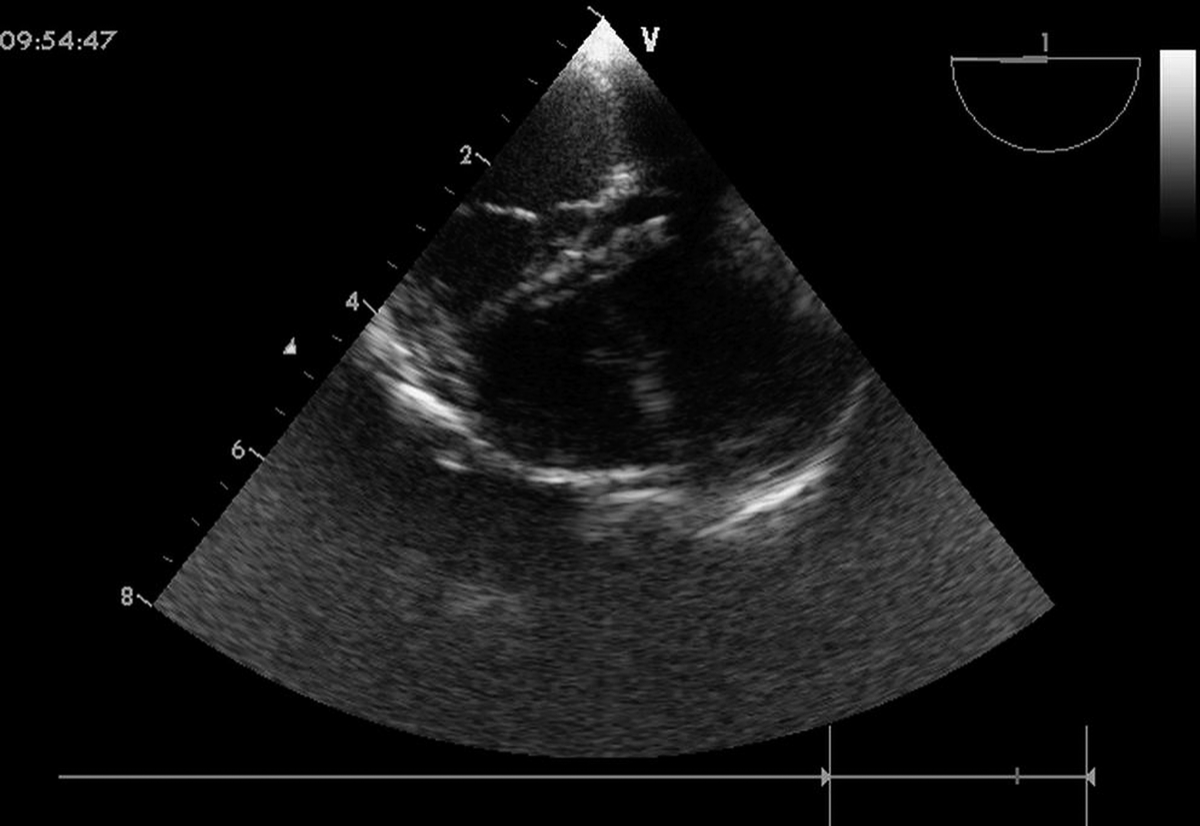
**A sheath was advanced over the wire into the left ventricle across the VSD**.

**Figure 4:**
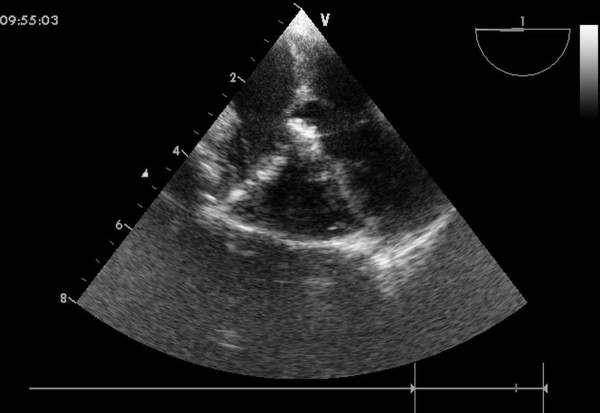
**The left disc was deployed**.

**Figure 5:**
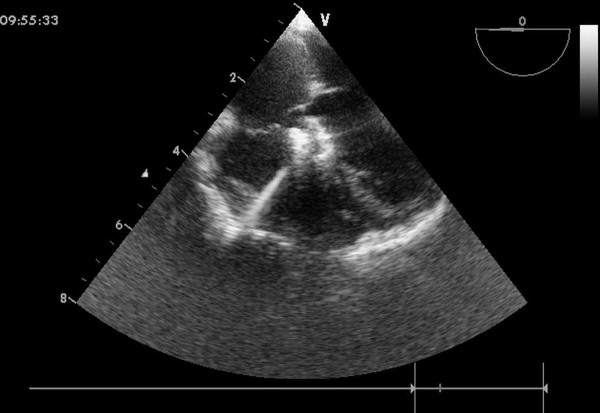
**Final image showed after a device was deployed**.

An inferior median sternotomy was created (3-5 cm) and a small rib spreader was used to manipulate the incision and facilitate instrumentation. The pericardium was opened and suspended to expose the right ventricle. On the free wall of the right ventricle two parallel 5-0 or 4-0 prolene sutures were placed and 1 mg/kg IV heparin was administered to maintain an activated clotting time > 250 sec. A modified short angiocatheter was passed into the right ventricle through the free wall and then the needle was removed. A floppy wire was advanced through the angiocatheter in the direction of the perimembranous VSD (Figure 2). The VSD was crossed and the wire was advanced through the defect into the left ventricle. The dilator was removed, and an appropriately sized delivery sheath was advanced over the wire into the left ventricle. The wire and the dilator were removed, and the sheath was allowed to back bleed to ensure there was no air entrapment (Figure 3). The VSD occluder was immersed in saline and screwed onto the delivery cable. The device was loaded with a loading sheath, which was subsequently introduced into the delivery sheath. The occluder was advanced to the tip of the sheath, and the sheath was gently pulled back until the tip was in the left ventricle. Under echocardiographic guidance, the left disc was deployed and the sheath was pulled back slowly until the left disc approximated the ventricular septum (Figure 4). To implant the asymmetric device, the device was gently rotated so the platinum marker of the distal disk pointed down and the flat part of the disc was not directly under the aortic valve. The waist and the right disc of the occluding device were deployed while maintaining traction on the delivery cable (Figure 5). TEE was repeated to ensure no significant residual shunt or aortic or tricuspid valve regurgitation. The occluder was then released by rotating the delivery cable counterclockwise. The sheath and the delivery cable were withdrawn. Closure was routine, and no drainage tube was necessary. Oral dipyridamole or aspirin were prescribed for 3-6 months for anticoagulation [11].

The present study was approved by the ethics committee of the authors' university and adhered to the tenets of the Declaration of Helsinki. Additionally, the written informed consent was obtained from the patients or their parents.

### Statistical analysis

Continuous data were presented as mean ± standard deviation and range.

## Results

In total, 97 patients (42 males) underwent intraoperative device closure treatment. Mean patient age was 13.2 ± 11.8 years (range, 1-56 years) and mean body weight was 34.1 ± 16.9 kg (range, 8.5-70 kg). Delivery of the occluder was successful in 94 patients. Mean size of the perimembranous VSD measured by TEE was 7.6 ± 1.6 mm (range, 5-12 mm). Mean size of the implanted occluders was 8.8 ± 1.9 mm (range, 6-14 mm) and the diameter of the sheath ranged from 6-9 F.

Intraoperatively, complete AVB occurred in one patient and a Mobitz type II AVB occurred in one patient. In both patients, the AVB resolved quickly after discontinuing the procedure. In addition, two patients developed Mobitz type II AVB intraoperatively. Because those two patients intermittently recovered sinus rhythm, temporary pacemakers were inserted. After the administration of glucocorticoids, the two patients with Mobitz type II AVB converted to Mobitz type I AVB in 3 and 5 days.

Immediately postprocedurally, Mobitz type I AVB was observed in two patients. No intervention was required, but patients were closely observed. One patient presented with complete AVB 1 week after device insertion. Surgical device removal was followed by a rapid and complete recovery of atrioventricular conduction.

In addition to the AVBs noted above, minor transient arrhythmias were encountered in 12 patients during the course of device deployment, including temporary sinus bradycardia, atrial premature beats, and transient left bundle branch block. All of those minor arrhythmias either spontaneously resolved or were easily and successfully treated pharmacologically.

The mean and median follow-up period was 1.37 ± 0.56 years and 1 year, respectively (range, 6 months to 2 years) for patients in which the occluders were successfully implanted. Outpatient follow-up was achieved with functional, echocardiographic, and electrocardiographic assessment. No progressive arrhythmias were noted during the follow-up period. To date, none of the patients in the study cohort developed complete AVB.

## Discussion and Conclusions

VSD is one of the most common congenital cardiac defects. Elective open-heart repair with midline sternotomy and cardiopulmonary bypass has been considered the gold standard for the closure of the perimembranous VSD; however, with the advent of various devices and techniques, percutaneous transcatheter occlusion of the VSD has gradually become a viable option for select patients. The safety and feasibility of transcatheter closure of a perimembranous VSD using Amplatzer septal occluders in many centers has been demonstrated, and complications seem to be limited [12-15]. At the authors' institute, a hybrid method (described by Xing Quansheng and his colleagues') is employed to close perimembranous VSDs, which includes the use of an intraoperative device and minimally invasive approach [16,17]. Similar to other reports of transcatheter closure of perimembranous VSDs, we have also achieved high technical success and good outcomes. Due to the close proximity of the perimembranous VSD to the conduction system, however, one complication that can occur is AVB. Recently, an increasing number of studies have begun to focus on this complication.

Complete AVB is an uncommon complication of congenital heart disease surgery. According to Tucker, operative AVB and permanent pacemaker placement occurred in 1.1% and 0.8% of their patients, respectively, which is comparable to those currently considered for device closure of the perimembranous VSD. A permanent pacemaker is reportedly more likely in patients with Down syndrome and in younger patients [18].

Andersen and his colleagues reported that iatrogenic complete AVB continued to occur after surgical VSD closure either because of unexpected biological variations or because of a lack of awareness of the disposition of the atrioventricular conduction axis in particular circumstances. That report addresses the atrioventricular conduction axis and suggests that the risk of iatrogenic complete AVB is < 1% [19]. Although not common, surgeons' awareness of this potential complication and increased experience performing the surgical technique likely have reduced the incidence of complete AVB. Pacemaker insertion is usually recommended if there is no recovery of the atrioventricular conduction either intra- or postoperatively. As opposed to complete AVBs that occur intraoperatively, complete AVBs can occur days, weeks, or even months later following device deployment. Sporadic studies have reported the incidence of transient complete AVBs in up to 1-5% of patients undergoing transcatheter device closure of perimembranous VSDs. For example, Zhou et al. reported the incidence of AVB occurring either during or after transcatheter closure of perimembranous VSDs was 3.5% in a series of 168 patients that included a 6-24 month follow-up period [9]. In a study by Butera and colleagues, two patients (2.5 years and 4 years of age) treated with eccentric VSD occluder devices developed complete AVB that was diagnosed 4 and 12 months postprocedurally. Both subjects underwent endocardial ventricular pacemaker implantation [7]. Predescu and colleagues reported that acute complete shunt occlusion was achieved in all patients, but during the median follow-up period of 23.1 months (range, 1-37.8 months), four patients (22%) had complete AVB 17 days, 4.2 months, 8.8 months, and 37.5 months after implantation. No risk factors were identified for development of complete AVB. Thus, although that study reported a high technical success with 100% shunts ultimately occluded, an unacceptably high rate of complete AVB occurred. Based on that data, perimembranous VSD device implantation was terminated at that medical institution [10]. Compared with Predescu et al.'s data, the incidence of complete AVB was only 2.1% in the current study.

Early AVB has been relatively widely studied in the past; however, the exact underlying mechanism(s) of AVB remain(s) speculative. It is in the perimembranous ventricular septal area that the atrioventricular conduction bundle emerges from the central fibrous body to become subendocardial. Thus, due to its anatomic location, the atrioventricular conduction bundle may be prone to damage during correction of VSDs. Conduction system injury from mechanical trauma/compression by either the delivery system or the device itself can potentially cause acute intraprocedural complete AVB. Some cases of early complete AVB were resolved spontaneously, with short-term corticosteroid therapy, or device removal [8,9,20]. In the authors' experience, complete AVB occurred in only one case (in addition to the one Mobitz type II AVB) during the procedure. In both of those cases the intraoperative AVBs disappeared quickly after discontinuing the procedure. Despite the limited number of AVB cases, these results have led the authors to believe that AVB occurring immediately after device occlusion may result directly from mechanical trauma/compression to the conduction system. The authors recommended abandoning the procedure if complete AVB occurs, ensuring that the defect is carefully traversed with the delivery sheath. Careful manipulation of devices when crossing the defect is likely imperative to avoid AVB.

In addition, the surgical approach described herein provides cardiac surgeons with a perpendicular angle to the perimembranous ventricular septum, which may facilitate guiding the wire through the VSD and deploying the occluder into the defect without causing any trauma to the atrioventricular conduction pathway. This is evidenced by the small number of patients who developed AVBs in this study. The size and position of the device must be carefully verified via TEE both before and after deployment. The selected device should be either the same size or only 1-2 mm larger than the VSD (as determined by the TEE). From the little evidence that we have to date, the use of an oversized device should be avoided.

One study has suggested that smaller patients might be at higher risk for AVB than older patients [10]. In the current study, the one case of complete AVB that occurred Intraoperatively was a 4 year old boy. His VSD measured 5 mm in diameter and a 6 mm occluder was deployed in this case. Clearly, age is not likely the only issue and additional risk factors need to be identified. Indeed, it remains unclear why certain patients develop complete AVB and others do not and why the AVB occurs intraoperatively in some patients but either early or late in the postoperative period in others. It is possible that AVBs that occur weeks to months after device deployment could be due to inflammation and fibrosis. Occluders have been shown to cause a localized inflammatory reaction that can result in extensive scar tissue and cartilaginous metaplasia of the surrounding myocardium [6]. A number of studies have reported on the late onset of complete AVB [7,21]. Chen for example report one case of delayed complete AVB with severe tricuspid regurgitation occurring 5 years after implantation of an eccentric Amplatzer perimembranous VSD occluder [22]. Similarly, Yalonetsky et al. presented a patient who developed symptomatic Mobitz type II AVB 3 years following percutaneous closure of such a defect [23]. Device flattening or shifting is another mechanism that may be responsible for some of the late instances of AVB. In the current study, a 6 year old boy treated with an 8 mm occluder developed complete AVB 1 week postprocedurally. Device removal together with surgical VSD closure was immediately performed, followed by a rapid and complete recovery of normal atrioventricular conduction. During the surgical closure, the right ventricular disc was found impinging on the septum. It is possible that this device shift disturbed the atrioventricular conduction by direct traumatic compression. Patients and their parents should therefore be informed regarding the risk of sudden, potentially life-threatening late-onset complete AVB. Long-term follow-up, perhaps life-long follow-up, may be indicated (or mandatory) in patients that have had occluders implanted for VSDs. Based on this study, the chances of developing delayed-onset complete AVB are the same regardless of the surgical approach.

Although it is not clear how AVB can be avoided in patients with VSDs, the authors recommended abandoning the procedure if complete AVB occurs. Some authors suggest that a course of steroids may be useful in reversing AVB [6,8], but the effectiveness of these drugs has yet to be determined. Nonetheless, we administered corticosteroids to the two patients with Mobitz type II AVB. These two patients recovered sinus rhythm intermittently during the operation, so temporary pacemakers were inserted in these patients. These two patients subsequently improved (to Mobitz type I AVB) in 3 and 5 days. Implantation of permanent pacemakers and timing of implantation remain controversial topics. In our opinion, surgical closure remains the best treatment of choice for patients who present with complete AVB.

This single-center experience included only a small number of patients. Nonetheless, this study notes that complete AVB can occur either during the course of device closure of the perimembranous VSDs or postoperatively. This study is not, however, representative of the entire perimembranous VSD device experience, which includes many older and larger patients and smaller defects. All of the VSDs in this study were restrictive. It should also be noted that this study was conducted in a low-income region where health care resources are limited. Although device closure of perimembranous VSDs appear to be safe in the short-term, it remains unknown whether they are safe in the very long-term. Given these circumstances, longer follow-up data are needed.

In conclusion, this study demonstrates that intraoperative device closure of perimembranous VSDs is safe and feasible based on short-term follow-up data. Complete AVB is a serious complication that can occur both during and after device closure. No risk factors for complete AVB were identified in this study. Additional multi-center studies are needed, and long-term follow-up is essential for all patients who have had devices implanted.

## Competing interests

The authors declare that they have no competing interests.

## Authors' contributions

QC designed the study, collected the clinical data and performed the statistical analysis, participated in the operation and drafted the manuscript. HC, G-cZ and L-wC participated in the operation and revised the paper. Q-zL and Z-hQ participated in the operation and collected the clinical data. All authors read and approved the final manuscript.

## Pre-publication history

The pre-publication history for this paper can be accessed here:

http://www.biomedcentral.com/1471-2261/12/21/prepub
